# Potential distribution prediction of *Ceracris kiangsu* Tsai in China

**DOI:** 10.1038/s41598-024-64108-2

**Published:** 2024-06-11

**Authors:** Chun Fu, Xuanye Wen, Zhaopeng Shi, Lin Rui, Na Jiang, Gelin Zhao, Rulin Wang, Jinpeng Zhao, YaoJun Yang

**Affiliations:** 1 Key Laboratory of Sichuan Province for Bamboo Pests Control and Resource Development, Leshan Normal University, Leshan, 614000 People’s Republic of China; 2 Center for Biological Disaster Prevention and Control, National Forestry and Grassland Administration, Shenyang, 110031 People’s Republic of China; 3 Extension Center for Agricultural Technology of Shandong Province, Jinan, 250100 People’s Republic of China; 4 Cangxi Meteorological Bureau, Cangxi, People’s Republic of China; 5 College of Tourism and Geographical Science, Leshan Normal University, Leshan, 614000 People’s Republic of China; 6 Mianyang Teachers’ College, Mianyang, 621000 People’s Republic of China; 7 Sichuan Provincial Rural Economic Information Center, Chengdu, 610031 People’s Republic of China

**Keywords:** Ecology, Ecology

## Abstract

*Ceracris kiangsu* Tsai (*C. kiangs*) is a kind of forest pest, which can harm nearly 100 kinds of weeds and crops. In this study, based on 314 species distribution points of *C. kiangsu* which were obtained from Chinese herbaria, literatures and investigation, and data of three future climate scenarios presented by CMIP6, two niche models (Garp, Maxent) were used to predict the suitable area of *C. kiangsu* in China. The result shows that the main environmental factors affecting the distribution of *C. kiangsu* are precipitation of driest month (bio14) and min temperature of coldest month (bio6). No matter now and future, the potential distribution areas of *C. kiangsu* in China are mainly in the south of Qinling–Huaihe River. Under current scenarios, the areas of the total, highly, moderately and poorly suitable of *C. kiangsu* in China are 160.65 × 10^4^ km^2^, 31.70 × 10^4^ km^2^, 60.36 × 10^4^ km^2^ and 68.59 × 10^4^ km^2^ respectively. The southern Hubei, western Jiangxi and eastern Hunan are highly-suitable areas. Under SSP1-2.6 and SSP2-4.5 scenarios, both the total suitable area and the highly suitable show a decreasing tread in 2050s. Compared to the 2050s, the total suitable area will coninue to decease in 2090s under SSP1-2.6, while it will increase under SSP2-4.5. The highly suitable area will increase in both scenarios, and the increased percentage under SSP2-4.5 is greater than that under SSP1-2.6. Under SSP5-8.5 scenarios, the total suitable area will increase by 1.83% in the 2050s, and decrease by 1.17% in the 2090s. The highly suitable area in the 2050s and 2090s under this scenarios is larger than under current scenarios. No matter what the scenario, the southern part of Yunnan, the southeast of Sichuan and the southwest of Chongqing will become highly-suitable areas as the climate continues to warm and should be monitored more cosely.

## Introduction

*Ceracris kiangsu* Tsai belongs to Orthopera Arcypteridae, nymphs or adults feed on leaves and shoots. *C. kiangsu* is distributed in southern China, Vietnam, Laos and other tropical and subtropical areas^1–3^, which can harm nearly 100 kinds of weeds and crops, such as *Phyllostachys*, *Bambusa*, *Zea mays*, *Oryza sativa* and *Trachycarpus fortunei*^4,5^. *C. kiangsu* is seriously harmful to bamboo industry, it has a wide variety of food, a large amount of food, a long time of harm, and is difficult to control. When the locust plague is severe, *C. kiangsu* can eat up the leaves of the bamboo forest, resulting in the death of the young bamboo, and new bamboo will not grow for 2–3 years after the disaster^6^.

*Ceracris kiangsu* is one of the earliest recorded forest pests in our country, which can be traced back to the Jiaqing period of the Ming Dynasty^7^. In recent years, with the continuous expansion of the national economic forest area, the harm of *C. kiangsu* has risen again. Since June 2020, the migration of overseas *C. kiangsu* along the China-Laos border in Yunnan Province has caused harm to agricultural and forestry production in relevant areas, which has attracted great attention of the Ministry of Agriculture and Rural Affairs, and the National Forestry and Grassland Administration. Recently, some scholars have carried out a lot of research on the *C. kiangsu*, mainly focuses on biology and control technology^8,9^, but the research on its potential distribution in China is still less. Therefore, it is of great significance to predict and analyze the potential distribution of *C. kiangsu* in China for disaster prevention and risk assessment.

Niche model is a method to describe the ecological needs of species and analyze the suitability of species by using mathematical model and spatial projection through species distribution and environmental variables. It has been applied to the distribution of rare plants^10^, the alien species invasion^11^, wildlife protection^12^ and other fields, which has achieved good results. According to relevant studies, Maxent model and Garp model are the most widely used in common species distribution prediction software^13^. Based on the known spatial distribution, MaxEnt (Maximum Entropy Model) model searches for key environmental factors, builds constraint sets, and simulates the relationship between spatial distribution and environmental factors. The MaxEnt model is widely used to predict the suitable areas of forest pests, such as *Bemisia tabaci*^14^, *Locusta migratoria tibetensis*^15^, *Batocera horsfieldi*^16^ and so on. GARP (Genetic Algorithm for Rule-set Production) is a rule-based niche modeling method, which can consider the effects of multiple environmental factors and habitat complexity on species distribution. The model can be used to study niche differentiation and biological invasion^17,18^, which can generate rule sets that conform to ecological laws and improve the accuracy of prediction.

CMIP6 is a new phase of the Coupled Model Intercomparison Project launching by a new phase of the Coupled Model Intercomparison Project, which aims to address new scientific questions in climate change field and provide data support for the scientific goals established by the WCRP Grand Challenges program^19^. According Jin’s research, ACCESS–CM2, CMCC–CM2–SR5 and CMCC–ESM2 have the best performance in the comprehensive simulation ability of temperature in Southwest China among the 19 future climate scenarios from CMIP6^20^. In this study, we chose the ACCESS–CM2 model developed by Australian scientists.

In this paper, the key environmental factors affecting the survival of *C. kiangsu* were analyzed by knife-cutting method, and the model (MaxEnt and Garp) combined with ArcGIS were used to predict the potential suitable area of the *C. kiangsu* in China under the current and future climatic scenarios. The study aimed to provide a scientific reference for researching and developing the feasible countermeasures against *C. kiangsu*, reduce the serious economic loss caused by it, and ensure the steady development of bamboo and crops industry in China.

## Results

### Model accuracy evaluation

The MaxEnt and Grap were tested for accuracy, and the results of training set and test set repeated 10 times were obtained (Table 1). The mean AUC values of two niche models (Maxent and Garp) were 0.979 and 0.921, and the mean standard error were 0.005 and 0.004, indicating that these two models have significant consistency and high prediction accuracy, and the existing distribution data and environmental factors can effectively predict the potential suitable area of *C. kiangsu* in China.

**Table 1:** The AUC values of two models.

	1	2	3	4	5	6	7	8	9	10	Average
Maxent	0.975	0.976	0.982	0.987	0.974	0.985	0.967	0.981	0.976	0.985	0.979
Garp	0.921	0.924	0.921	0.917	0.931	0.924	0.916	0.902	0.928	0.923	0.921

### Potential suitable of *C. kiangsu* in China

**Figure 1:**
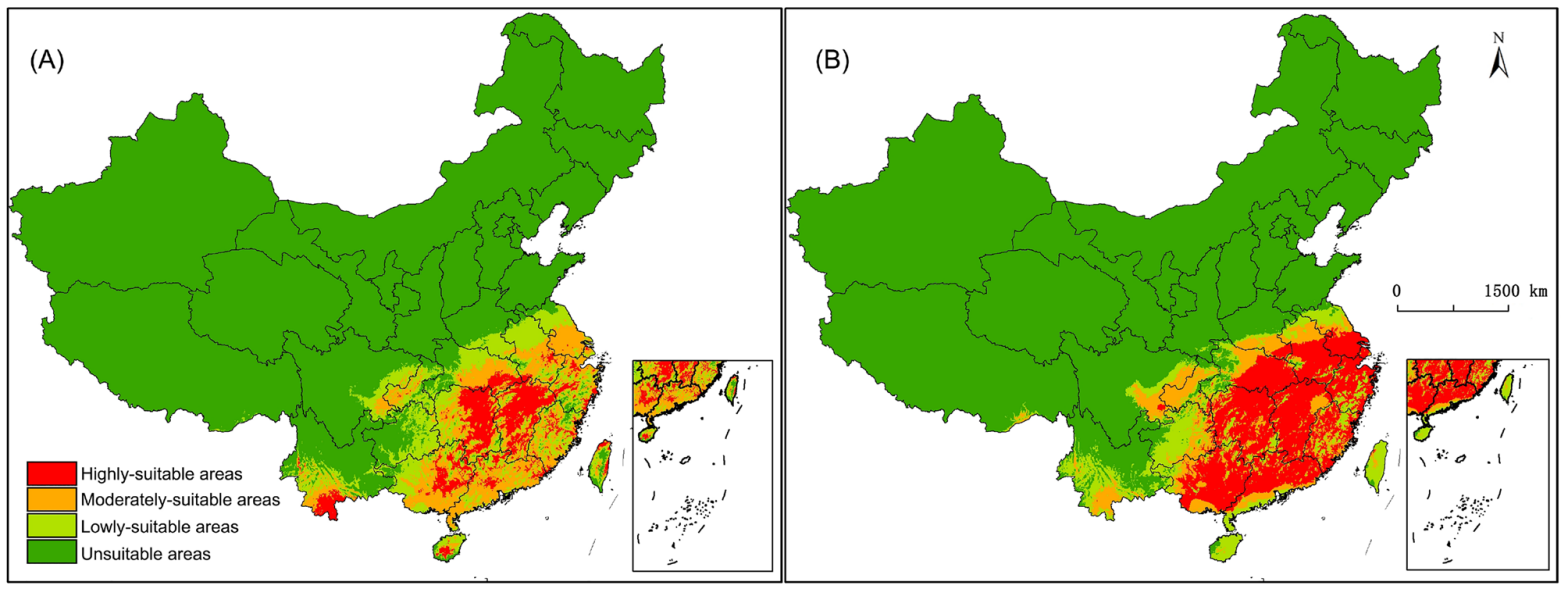
Predicted potential suitable habitats of *Ceracris kiangsu* under current scenarios by MaxEnt (**A**) and Garp (**B**). MaxEnt v3.4.4: https://biodiversityinformatics.amnh.org/open_source/maxent/, ArcGIS v10.0: https://www.arcgis.com/.

Under current scenario, the MaxEnt model (Fig. 1A) and Garp model (Fig. 1B) predict that the suitable areas of *C. kiangsu* are mainly distributed in the Yangtze-Huaihe river basin, the lower-middle reaches of the Yangtze River, South China and Southwest China, but the degrees of suitable areas are different. Figure 1A shows that the suitable areas account for 16.71% of the total land area in China, of which the highly-suitable areas account for 3.3%, the moderately-suitable areas account for 6.28%, and the lowly-suitable areas account for 7.13%. Figure 1B shows that the suitable areas account for 18.73% of the total land area in China, of which the highly-suitable areas account for 8.77%, the moderately-suitable areas account for 4.88%, and the lowly-suitable areas account for 5.08%. Compare the prediction result of the MaxEnt model and the Garp model, the suitable areas are all mainly distributed in Jiangxi, Hunan, Zhejiang and Anhui in the lower-middle reaches of the Yangtze River, Guangdong and Guangxi along the coast of South China and Yunnan in the southwest, but the different grades of area contain in each province varies greatly. The highly-suitable areas predicted by the MaxEnt model are divergent distribution with taking as the center of Poyang Lake plain and Lianghu plain in Xiang-gan area, while the Garp model’s are from the Guangzhou-Guangxi region to the Eo-Wan region. The moderately-suitable areas predicted by the MaxEnt model are mainly distributed in the centre of Anhui, the northwest of Hubei, the west of Hunan, most of Guangxi, the northwest of Chongqing and the southwest of Yunnan, while the Garp model’s are mainly distributed in the southern foot of Dabie Mountains in Eo-Wan region and the parallel ridge valley in eastern Sichuan.

**Figure 2:**
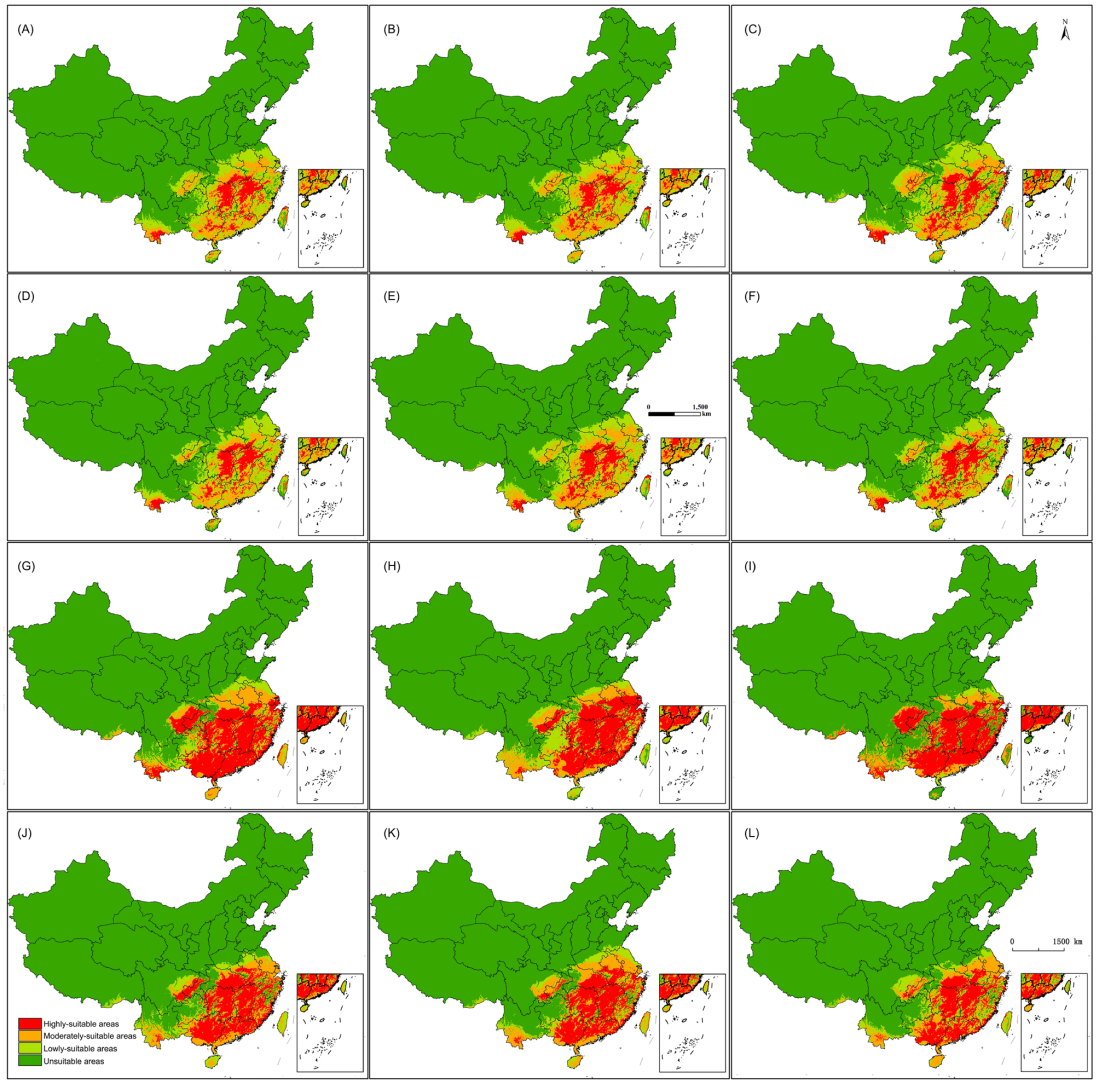
Predicted potential suitable habitats of *Ceracris kiangsu* under future scenarios by MaxEnt (**A**–**F**) and Garp (**G**–**L**). MaxEnt v3.4.4: https://biodiversityinformatics.amnh.org/open_source/maxent/, ArcGIS v10.0: https://www.arcgis.com/ (Note: A, G: SSP1-2.6, 2050s; B, H: SSP2-4.5, 2050s; C, I: SSP5-8.5, 2050s; D, J: SSP1-2.6, 2090s; E, K: SSP2-4.5, 2090s; E, L: SSP5-8.5, 2090s).

Compared with the current scenario, the MaxEnt model (Fig. 2A,D) predicts the suitable areas of *C. kiangsu* will decrease 0.26% by 2050s and 1.52% by 2090s under SSP1-2.6 scenario. The highly-suitable areas and the moderately-suitable areas will decrease the most, while the lowly-suitable areas will increase slightly. The Garp model (Fig. 2G,J) predicts the suitable areas of *C. kiangsu* will increase 1.54% by 2050s and then decrease 2.73% by 2090s. The highly-suitable area is the areas with the most obvious change, while the moderately-suitable area is relatively small change. The MaxEnt model (Fig. 2B,E) predicts the suitable areas of *C. kiangsu* will decrease 0.08% by 2050s and then increase 0.58% by 2090s under SSP2-4.5 scenario. From now to 2050s, The highly-suitable areas will decrease the most and the lowly-suitable areas will increase the most, while they will change very slightly from 2050 to 2090s. The Garp model (Fig. 2H,K) predicts the suitable areas of *C. kiangsu* will increase 1.74% by 2050s and then decrease 2.7% by 2090s. From now to 2050s, the highly-suitable area and the moderately-suitable area are the areas with the most obvious change, while the lowly-suitable area is relatively small change, only 0.14%. From 2050 to 2090s, the highly-suitable area and the lowly-suitable area are the areas with the most obvious decrease, while the lowly-suitable area is relatively small increase. The MaxEnt model (Fig. 2C,F) predicts the suitable areas of *C. kiangsu* will increase 1.83% by 2050s and then decrease 3.01% in 2090s under SSP5-8.5 scenario. From now to 2050s, The highly-suitable areas will decrease the most and the lowly-suitable area will decrease the most, while they will change very slightly from 2050 to 2090s. The Garp model (Fig. 2I,L) predicts the suitable areas of *C. kiangsu* will decrease 1.95% by 2050s and then decrease 0.3% in 2090s. From now to 2050s, the highly-suitable area and the moderately-suitable area are the areas with the most obvious change, while the lowly-suitable areas is relatively small change, only 0.14%. From 2050 to 2090s, the lowly-suitable area is the areas with the most obvious decrease, while the highly-suitable area and the moderately-suitable area are relatively small increase.

### Effects of environmental factors on the distribution of *C. kiangsu*

**Figure 3:**
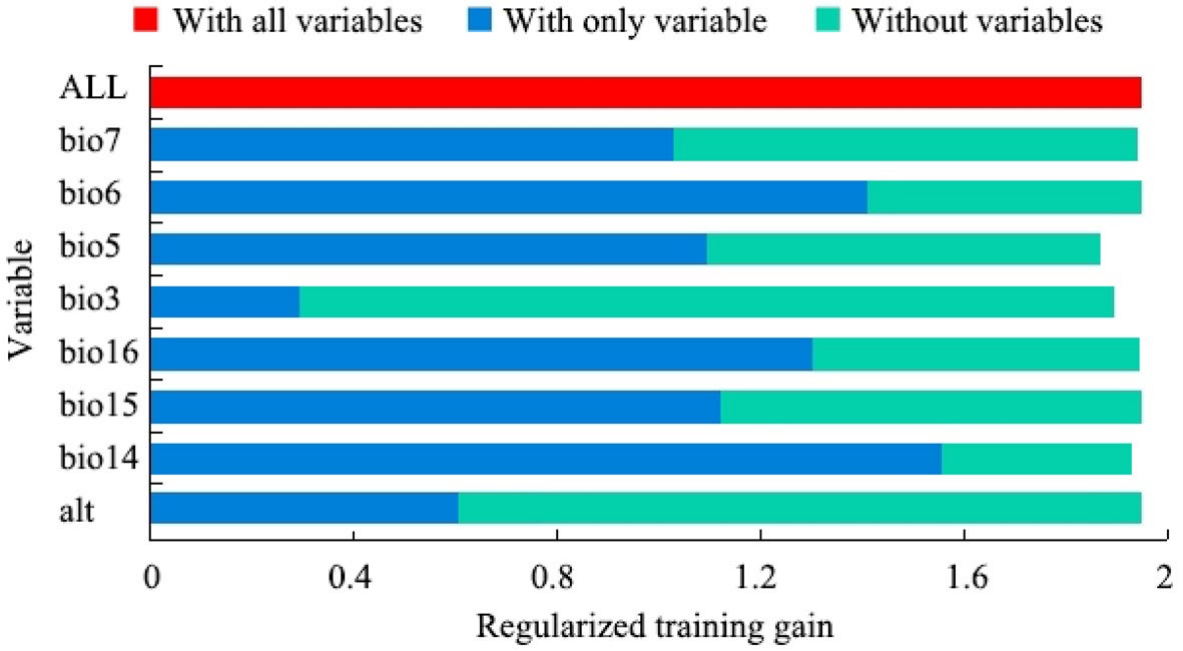
Jackknife test for evaluating the relative importance of environmental variables.

From the result of the jackknife, the contribution rates of precipitation of driest month (bio14) and min temperature of coldest month (bio6) are 51.5% and 22.3%, indicting bio14 and bio6 can provide more effective information in the modeling process. The regularized training gains of bio5, bio16, bio15 and bio7 are greater than 1.0 (Fig. 3), and the total contribution rate of these factors is 22.1%, indicting these factors are also important to the distribution of the *C. kiangsu.*

**Figure 4:**
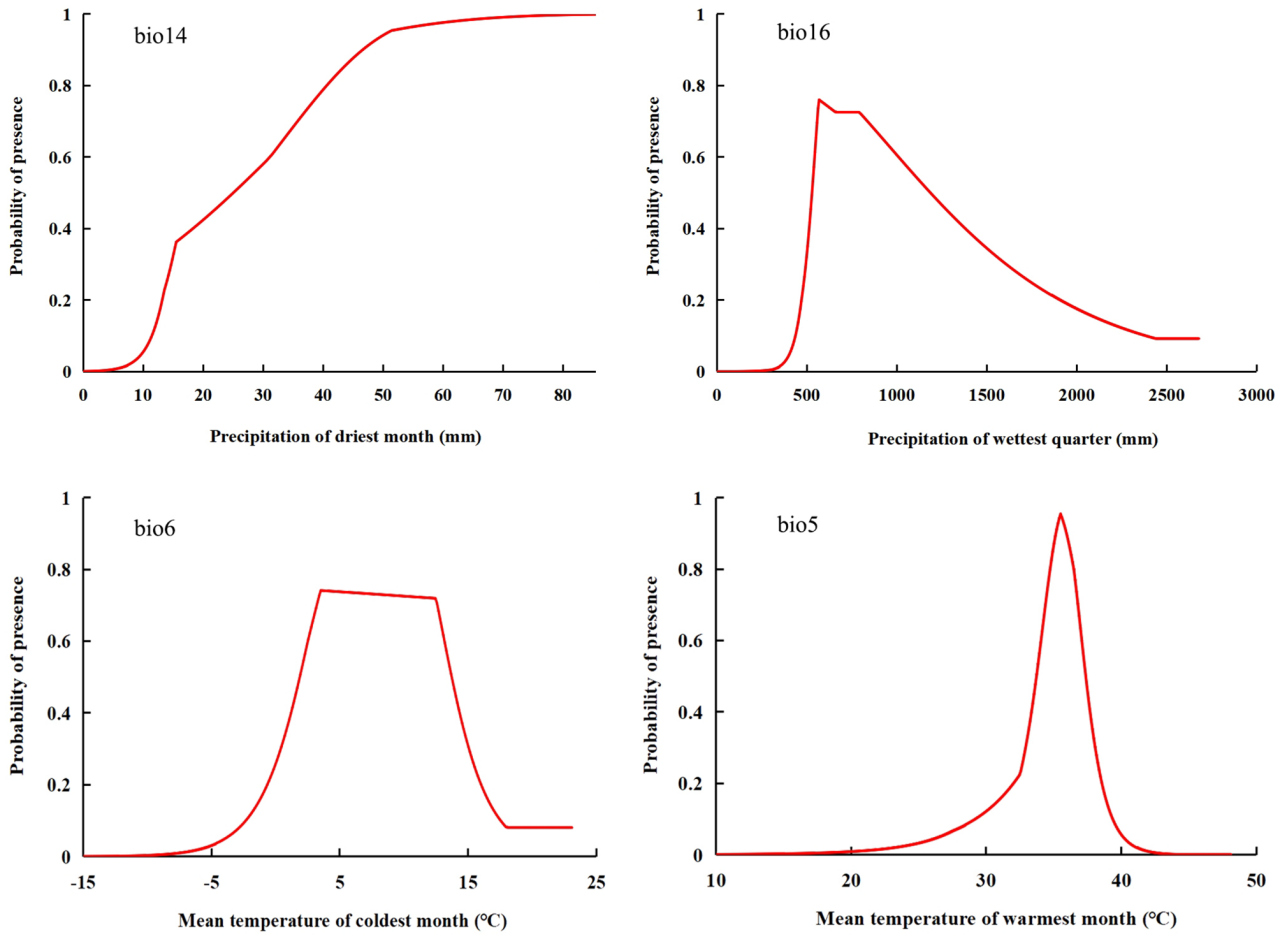
Relationship between different environmental variables and survival probability of *Cercris kiangsu.*

The main environmental variables affecting the distribution of *C. kiangsu* are precipitation and temperature. From Fig. 4, we can know when precipitation of driest month (bio14) is less than 15.3 mm, the survival rate of *C. kiangsu* is less than 0.33, and when the precipitation exceeds 34.2 mm, the survival rate rises to 0.66. According to the classification method of suitable areas, the variation range of precipitation of wettest quarter (bio16) is 498.2–1524.3 mm, and the optimum value is 568.5 mm. In terms of temperature, the optimum value of min temperature of coldest month (bio6) is 3.5 °C, and the survival rate of *C. kiangsu* will be reduced when the value of bio6 is lower or higher than this value. When max temperature of warmest month (bio5) is lower than 24.5 °C or higher than 38 °C, the survival rate of *C. kiangsu* is less than 0.33.

## Discussion

By comparison, it is find that Maxent model can fit the distribution of *C. kiangsu* in China well, which the average value of AUC are higher than Garp model. Relevant literature also proves that Maxent can predict the suitable area of species well under both large or small sample conditions^21^ The Garp model predicts a wide range of highly-suitable area for *C. kiangsu*, which is similar to what other scholars have done in predicting other species^22,23^. This may be due to two reasons, one is that the output type of Garp is boolean, and the result is suitable as long as species shows adaptation somewhere, another may be due to insufficient repetitive Settings, the classification is not obvious.

*Ceracris kiangsu* is host-oriented leaf-eating pests, and its growth and development are closely related to host species^24^, so its suitable areas are directly related to the host distribution. According to the 9th forest resources inventory in China, the main producing areas of bamboo resources in China are Hunan, Jiangxi, Fujian, Zhejiang, Anhui, Sichuan, Zhejiang and Guangdong^25^, which is basically consistent with the distribution results of *C. kiangsu* obtained in this study. From predict results of MaxEnt model and Garp model, the southern Hubei, the western Jiangxi and the eastern Hunan are highly suitable areas for *C. kiangsu*. This result is consistent with Yang’s^26^ research, and *C. kiangsu* are currently appearing in these places^27–29^. As the climate warms in the future scenarios, the southern part of Yunnan, the southeast of Sichuan and the southwest of Chongqing will become the highly-suitable areas. The local vegetation and climate conditions of these areas mentioned above are more suitable for the survival of *C. kiangsu*. It is recommended that the local forestry authorities strengthen the monitoring of bamboo forests from the meteorology and biology to prevent the damage caused by the introduction of *C. kiangsu*. The migration and breeding of *C. kiangsu* can be predicted by monitoring meteorological parameters such as temperature, humidity and wind direction and speed, while the populations of *C. kiangsu* can be known in time and countermeasures can be made in a timely by collecting and identifying eggs, nymphs and adults in the field,.

Global warming, especially the rise in winter temperature, is conducive to the increase of *C. kiangsu*’s overwintering eggs, which provide "eggs" for the outbreak of *C. kiangsu* in the next year. In addition, the combination of climate change, worsening drought and grassland degradation will provide suitable areas for *C. kiangsu* to lay eggs. So, the habitat boundary of *C. kiangsu* may be expand further north. But we can know from our research that the potential distribution of *C. kiangsu* in China is still in the south of Qinling-Huaihe River in future climate scenarios, which may indicate that the low temperature in winter in the northern region is till in the range that restricts the migration of *C. kiangsu* to the north, but the highly-suitable areas and the moderately-suitable areas in high latitude tend to be closer to the Qinling-Huaihe River, which plays a warning role in the control of *C. kiangsu.* To the new highly-suitable and moderately-suitable areas such as the southeast of Sichuan, the southwest of Chongqing and the south of Jiangsu, should be strengthened to quarantine inspection and pest monitoring in combination with the spatial and temporal distribution of bamboo. From the perspective of *C. kiangsu’s* suitable area in China under different future climate scenarios, the suitable area of *C. kiangsu* in China increased or remained unchanged in 2050s, but decreased in 2090s, which indicates that the continuous warming of climate is unfavorable to the survival of *C. kiangsu.*

Analysis of key environmental factors shows that the important factors affecting the distribution of *C. kiangsu* are precipitation of driest month (bio14), precipitation of wettest month (bio16), mean temperature of coldest month (bio6) and mean temperature of warmest month (bio5). June to August is the wettest month and the warmest month, and is the key period for adult emergence from the biological characteristics of *C. kiangsu*. At this time, insufficient precipitation will increase the risk of water loss of larva^30^, while excessive precipitation will increase the mortality of larva*.* Similarly, temperature within a certain range will promote its development, vice versa will inhibit. The coldest month and the driest month in South China is January, when it is winter. *C. kiangs* overwinters in the form of egg, and the winter temperatures too high or too low will damage embryo development and reduce hatching rates^31^.

The predict results of Maxent model and Garp model just represent the possibility of species distribution, and cannot completely represents the actual distribution of species. The potential distribution of *C. kiangsu* predicted in the paper was mainly based on 19 biological climate variables and an altitude factor, but it was not considered that the bamboo producing areas in China were mainly affected by the southwest monsoon in Yunnan and other places. In addition, the migration ability of *C. kiangsu* is very strong, so the air flow may be the key factor affecting its migration and spread. In the later stage, we will strengthen the research in this area.

## Materials and methods

### Sample distribution data

**Figure 5:**
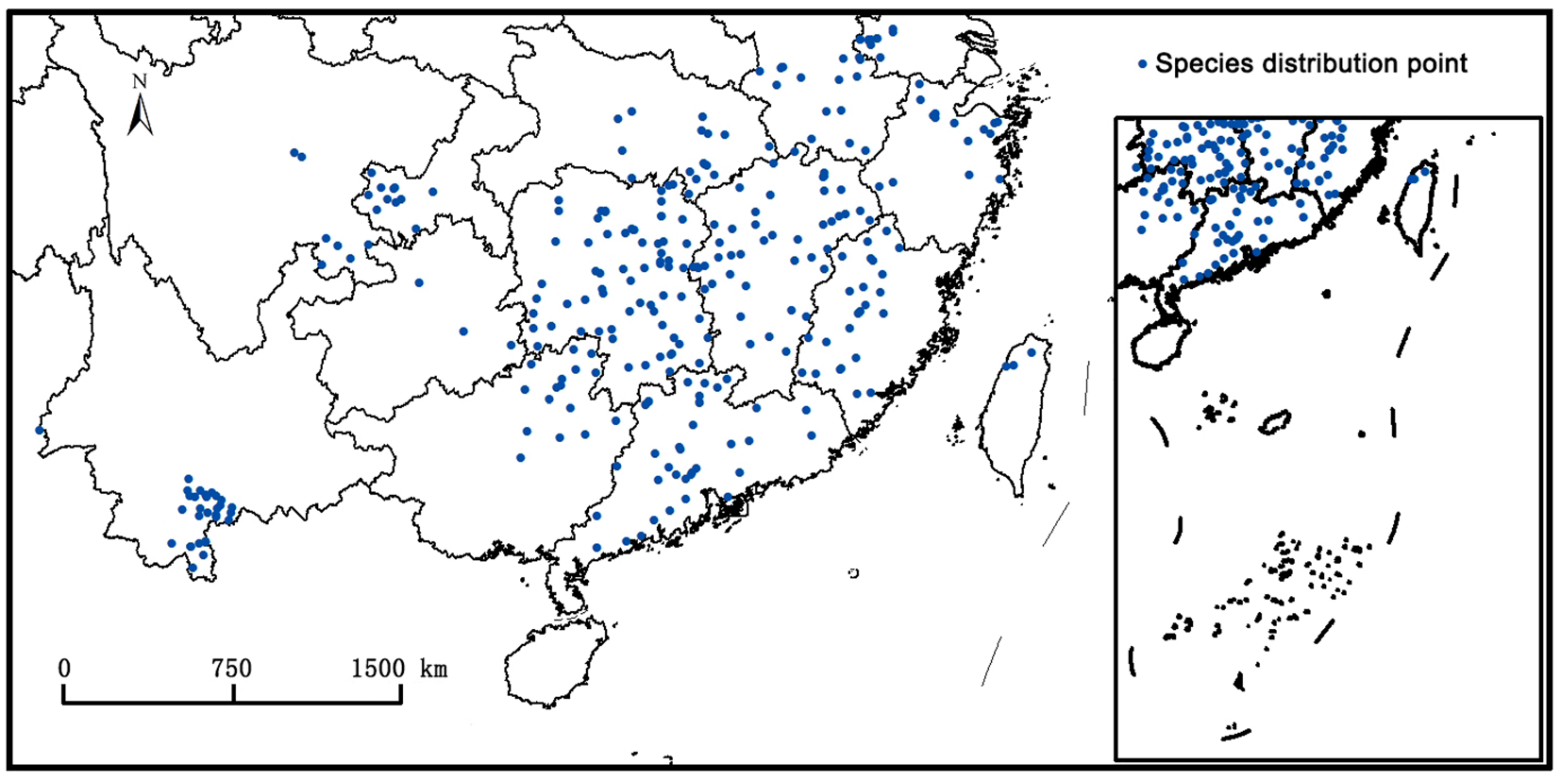
Distribution point of *Ceracris kiangsu* in China. ArcGIS v10.0: https://www.arcgis.com/.

The distribution data were obtained from the Global Biodiversity Information Facility (GBIF)^32^, Centre Agriculture Bioscience International (CABI)^33^, field investigation and literature^8,34–39^*.* For the species distribution records missing the latitude and longitude coordinates, Google Earth was used to retrieve them. In order to avoid the duplication of ecological spatial distribution points caused by excessive geographic spatial sampling^40^, the ENMTOOL software was used to remove duplicate points in the resolution grid of 2.5 arc-min, which can reduce the spatial deviation of sampling points. After the above steps, a total of 314 species distribution points were obtained, including 279 points from the GBIF and CABI, 21 points from field investigation and 14 points from literature (Fig. 5).

### Environmental factors used in models

Nineteen bioclimatic factors and elevation for historical periods downloaded from WorldClim (https://www.worldclim.org/) were selected as initial environmental factors. The jackknife method was used to test the contribution percentage of nineteen bioclimatic factors, and the factors with zero contribution rate were eliminated. In order to reduce the impact of multiple contributions of environmental factors on the model and avoid errors in modeling caused by spatial correlation, pearson collinearity test was conducted for nineteen bioclimatic factors with SPSS 14.0 software. When the correlation coefficient of the two bioclimatic factors was greater than 0.85, it indicates that there is a common relationship between the two factors, and the factor with a low percentage contribution value in the jackknife test was excluded. After the above steps, seven bioclimatic factors, such as isothermality (bio3), max temperature of warmest month (bio5), min temperature of coldest month (bio6), temperature annual range (bio7), precipitation of driest month (bio14), precipitation seasonality (bio15) and precipitation of wettest quarter (bio16), and elevation (alt) were remained. Then these factors for future periods were downloaded from WorldClim for further analysis.

### Evaluation of model accuracy

The accuracy of the model was evaluated by receiver operating curve (ROC). It takes specificity (false positive rate) as the X-axis and sensitivity (omission rate) as the Y-axis, and uses the area under ROC curve (AUC) to evaluate the accuracy of the model. When the AUC value is less than 0.7, the accuracy of the prediction results is poor; when the AUC value is less than 0.9 and greater than 0.7, the accuracy of the prediction results is general; when AUC value is greater than 0.9, the accuracy of prediction results is good^41^.

### Predictive models and parameter settings

The software to run the MaxEnt model was downloaded from the official website (https://biodiversityinformatics.amnh.org/open_source/maxent/). The MaxEnt model has two options, regularization multiplier (RM) and feature combinations (FCs), which can be adjusted to optimize the accuracy of the MaxEnt model. The FCs option contains five features: linear (L), quadratic (Q), hinge (H), product (P) and threshold (T). Referring to Guo^42^ and Zhao’s^43^ research, the RM step was set to 0.5 and ranges from 0.5 to 4. The FCs was set as L, LQ, H, LQH, LQHP and LQHPT, respectively. Therefore, 48 different combinations of RM and FCs were established and the combination with the smallest AICc value was selected as the optimal model. Meanwhile, the training set and the test set were set to 75% and 25% distribution points. The maximum number of iterations and the number of repetitions were set to 500 and 10 times, while the range limit of convergence was set to 10^−5^. The software to run the Garp model was downloaded from website (http://www.lifemapper.org). According to Peterson’s^44^ research, the maximum number of iterations and the number of repetitions were set to 1000 and 10 times, while the range limit of convergence was set to 10^−2^.

### Suitable area division and calculation

The potential distribution results obtained by MaxEnt model were input into ArcGIS to convert into raster form. According to the existence probability (*P*) generated by the model, the natural break point classification method (Jenks) was used to classify the suitable areas: the unsuitable area (0 ≤ *P* < 0.05), the lowly-suitable area (0.05 ≤ *P* < 0.33), the moderately-suitable area (0.33 ≤ *P* < 0.66), and the highly-suitable area (0.66 ≤ *P* < 1). Then, the distribution map of *C. kiangsu* in China was drawn by the reclassification function of ArcGIS, and the pixel number of different suitable area was counted by "attribute-symbol system-unique value", so that the area of each suitable region was obtained.

## Data Availability

All data included in this study are available upon request by contact with the corresponding author.
